# Residues in the Distal Heme Pocket of *Arabidopsis* Non-Symbiotic Hemoglobins: Implication for Nitrite Reductase Activity

**DOI:** 10.3390/ijms17050640

**Published:** 2016-04-28

**Authors:** Nitin Kumar, Alessandra Astegno, Jian Chen, Alejandro Giorgetti, Paola Dominici

**Affiliations:** 1 Hypertension & Vascular Research Division, Department of Internal Medicine, Henry Ford Hospital, Detroit, MI 48202, USA; 2 Department of Biotechnology, University of Verona, Strada Le Grazie 15, 37134 Verona, Italy; alessandra.astegno@univr.it (A.A.); jianchen0722@gmail.com (J.C.); alejandro.giorgetti@univr.it (A.G.); paola.dominici@univr.it (P.D.)

**Keywords:** *Arabidopsis* hemoglobins, nitrite reduction, distal cavity, hypoxia

## Abstract

It is well-established that plant hemoglobins (Hbs) are involved in nitric oxide (NO) metabolism via NO dioxygenase and/or nitrite reductase activity. The ferrous-deoxy *Arabidopsis* Hb1 and Hb2 (AHb1 and AHb2) have been shown to reduce nitrite to NO under hypoxia. Here, to test the hypothesis that a six- to five-coordinate heme iron transition might mediate the control of the nitrite reduction rate, we examined distal pocket mutants of AHb1 and AHb2 for nitrite reductase activity, NO production and spectroscopic features. Absorption spectra of AHbs distal histidine mutants showed that AHb1 mutant (H69L) is a stable pentacoordinate high-spin species in both ferrous and ferric states, whereas heme iron in AHb2 mutant (H66L) is hexacoordinated low-spin with Lys69 as the sixth ligand. The bimolecular rate constants for nitrite reduction to NO were 13.3 ± 0.40, 7.3 ± 0.5, 10.6 ± 0.8 and 171.90 ± 9.00 M^−1^·s^−1^ for AHb1, AHb2, AHb1 H69L and AHb2 H66L, respectively, at pH 7.4 and 25 °C. Consistent with the reductase activity, the amount of NO detected by chemiluminescence was significantly higher in the AHb2 H66L mutant. Our data indicate that nitrite reductase activity is determined not only by heme coordination, but also by a unique distal heme pocket in each AHb.

## 1. Introduction

Nitric oxide (NO) is a small gaseous molecule, with a short half-life of a few seconds [1]. NO plays an important role in plant growth and development, immune response, adaptation to biotic and abiotic stress, such as hypoxia, drought, temperature and pathogen attack [2,3,4,5,6]. NO can be produced in plants through oxidative or reductive mechanisms, which may involve enzymatic or non-enzymatic routes [5]. However, in plants the absence of a well-established route that generates NO, analogous to that in mammals, is a topic of intense discussion and still debatable.

Recently, nitrite reductase activity has been proposed for animals, cyanobacteria and non-symbiotic plant hemoglobin (nsHbs) based on their ability to reduce nitrite into NO [7,8,9,10]. This activity seems to be an intrinsic property of heme-containing globins. However, nsHbs from plants are described as being better nitrite reductases than animal Hbs [8,9], which is reasonable because this is how plants reduce the toxic effects of nitrite or nitrate accumulation during oxygen deprivation, flooding or waterlogging. The NO produced may undergo a downstream signaling pathway by modulating gene expression in response to stress conditions [11]. NO can also be converted to ammonia via hydroxylamine and contributes to nitrogen metabolism in plants [12]. It has been shown that NO released from nitrite by mammalian deoxy-Hb inhibits mitochondrial respiration and prevents the formation of both reactive nitrogen species (RNS) and reactive oxygen species (ROS) [7]. A similar mechanism might work in plants to prevent the formation of these highly reactive species. Three classes of nsHbs are found in *A. thaliana*, namely AHb1, AHb2 and AHb3. Class 1 nsHbs have a very high oxygen affinity (*K*_m_ ≈ 2 nM) [13,14], and their expression is highly induced under hypoxia and by the addition of either sucrose or nitrate [15,16]. Notwithstanding, the low concentrations found *in vivo* together with the small oxygen dissociation rate constants obtained would appear to imply that these nsHbs do not likely function in oxygen transport, while their high affinity for oxygen and redox potentials suggest that it is also improbable that they have an active role in electron transport [17,18]. *Arabidopsis* Hemoglobins 1 (AHb1) was reported to act as a NO scavenger participating in NO detoxification under hypoxic stress *in vitro* [19]. Considering *in vitro* data, the reaction of NO with oxyAHb1 may have physiological relevance in reducing the NO levels seen during hypoxic stress [19]. Class 2 nsHbs seem to be exclusive to dicots [20], and their gene expression is upregulated when plants experience low temperatures [15]. Moreover, they show tighter hexacoordination compared to class 1 nsHbs and have lower affinities for oxygen (*K*_m_ ≈ 100–200 nM) [21,22]. As a consequence, they are not as efficient in scavenging NO, while the possibility that they have a function that is related to sensing low levels of oxygen, as well as oxygen storage and diffusion becomes more probable [23]. Class 3 nsHbs are truncated and possess a very low similarity to class 1 and 2 nsHbs along with a low affinity for O_2_ (*K*_m_ ≈ 1500 nM) [24,25]. Differently from animal and symbiotic Hbs, class 1 and class 2 nsHb are hexacoordinated in both the ferric and ferrous state; this is related to the presence of a histidine in the distal pocket, which binds the sixth coordination site of the heme iron in the absence of external ligands in a reversible manner [13].

As previously reported, the UV-visible absorption spectra of the AHb proteins showed that ferrous-deoxy AHb2 wild type (wt) is completely hexacoordinated when exogenous ligands are not present, while ferrous-deoxy AHb1 wt is present as a combination of both hexa- and penta-coordinated species [26]. Carbon monoxide (CO) binding kinetics to AHb1 wt and AHb2 wt have shown remarkably different reactivity towards exogenous ligands of the two AHbs. Analogous to neuroglobin (Ngb) [27], a network of hydrophobic cavities that could temporarily store reactants and/or products was hypothesized to be crucial in sustaining the turnover of NO dioxygenase (NOD) activity in AHb1 [26,28,29]. Fourier transform infrared spectroscopy (FTIR) combined with temperature derivative spectroscopy has shown the presence of two CO docking sites in AHb1, while for AHb2, the same analysis has highlighted the absence of the secondary docking site for photodissociated CO [30].

It has been previously reported that, under conditions of hypoxia or anoxia, AHb1 and AHb2 can reduce nitrite into NO and, thus, function as nitrite reductases [9], with a reactivity similar to the reaction of nitrite with Hb, myoglobin (Mb) and Ngb [9,31]. In particular, in hexacoordinated Ngb, hexa-to-penta heme coordination significantly increases nitrite reductase activity [10]. However, the effect of hexa-to-penta heme coordination in nitrite reductase activity by AHb1 and AHb2 is not known.

To gain insight into this new function of AHbs, we have focused on the role of distal histidine (HisE7) and its protonation states in modulating the protein nitrite reactivity in both AHb1 and AHb2 through the study of the AHb1 H69L and AHb2 H66L mutants. Our data, in addition to confirming that under conditions of anoxia, *Arabidopsis* Hb1 and Hb2 can function as nitrite reductases, reveal that the unique distal heme pocket in each AHb together with the iron coordination are key determinants for nitrite reductase activity.

## 2. Results

### 2.1. Equilibrium Conformations of Arabidopsis Hemoglobins (AHbs) in the Absence of Distal His

**Figure 1:**
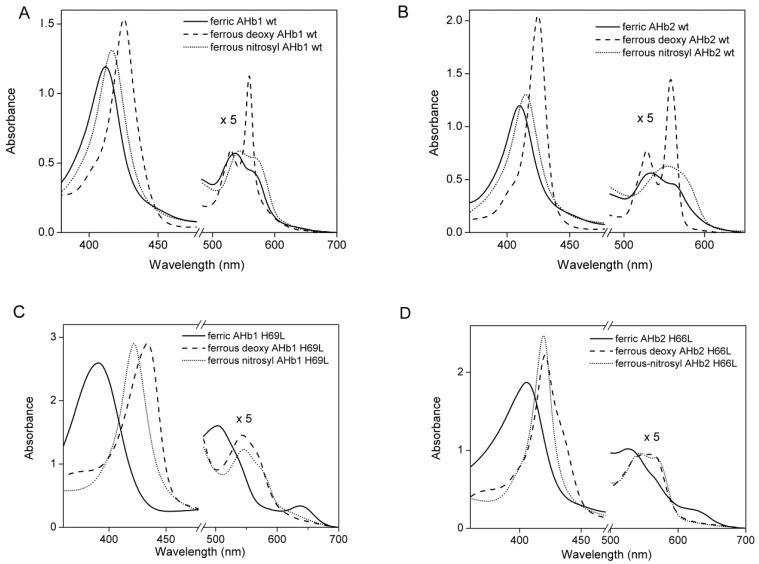
Reference spectra of AHbs wt and their distal histidine mutants. Absorption spectra of 20 µM ferric (solid line), ferrous-deoxy (dashed line) and ferrous-nitrosyl (short dotted line) (**A**) AHb1 wt, (**B**) AHb2 wt, (**C**) AHb1 H69L and (**D**) AHb2 H66L in 0.1 M phosphate buffer pH 7.4. The spectra are expanded by a factor of five from ~480 to 700 nm.

Reference spectra of AHb1 wt, AHb2 wt, AHb1 H69L and AHb2 H66L mutants in ferric, ferrous-deoxy and ferrous-nitrosyl forms are shown in Figure 1.

The UV-visible absorption spectra of the AHb proteins showed that deoxy AHb1 is present at a 40% fraction in a pentacoordinated state (Figure 1A), while ferrous-deoxy AHb2 is completely hexacoordinated when exogenous ligands are not present (Figure 1B) [26]. In the absence of His69 (E7) as the sixth ligand, the AHb1 mutant exhibited a pentacoordinate high spin iron both in the ferrous-deoxy form (which showed a red-shifted Soret band peaking at 435 nm) and in the ferric form (which showed a Soret band maximum at 390 nm and a visible region with a peak at 504 nm) (Figure 1C and Table 1). Moreover, an intense charge-transfer band was observed at 637 nm in the ferric form. The absorption spectra of both ferric and ferrous-deoxy AHb1 H69L were pH-insensitive in the range pH 6–10. All of these features clearly indicate that the iron in AHb1 H69L mutant is pentacoordinate high-spin, as expected for such a mutant.

**Figure 2:**
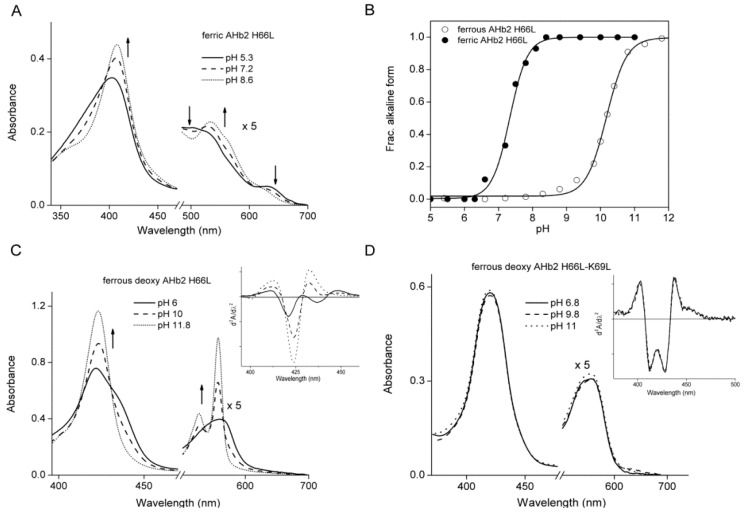
pH dependence of the absorption selected spectra of AHb2 H66L and AHb2 H66L-K69L mutants. (**A**) AHb2 H66L ferric form, spectra expanded by a factor of five from 480 to 700 nm. The up and downward pointing arrows indicate the increase and decrease in the spectral bands of AHb2 H66L with increasing pH; (**B**) absorption changes in the spectra of the AHb2 H66L mutant. Closed symbols, ferric form at 411 nm; open symbols, ferrous-deoxy form at 558 nm. The ordinate is scaled such that it represents the fraction of the deprotonated species. Data were fitted using Equation (3) shown in the Materials and Methods Section; (**C**) AHb2 H66L ferrous-deoxy form; spectra expanded by a factor of five from 500 to 700 nm. Inset, second derivatives of the ferrous-deoxy spectra in (**C**) from 395 to 460 nm at pH 6 (solid line), 10 (dashed line) and 11.8 (short dotted line). The arrows indicate change in the absorbance with increasing pH from 6 to 11.8; (**D**) spectra of ferrous-deoxy AHb2 H66L-K69L double mutant, expanded by a factor of five from 500 to 700 nm. Inset, second derivatives of the ferrous-deoxy spectra in (**D**) from 395 to 500 nm at pH 6.8 (solid line), 9.8 (dashed line) and 11 (short dotted line).

In contrast, heme iron in the AHb2 H66L mutant is hexacoordinated low-spin (Figure 1D and Table 1). Replacing His66 (E7) by leucine in AHb2 resulted in substantial pH-dependent spectral changes in both the ferric and ferrous-deoxy forms (Figure 2A, C and Table 1). The spectral features of the ferric AHb2 H66L mutant were similar to those of Mb wt at neutral pH. Mb contains a six-coordinate high-spin heme in the ferric state with a molecule of water as the sixth ligand of the heme iron [32].

To identify the sixth ligand in AHb2 H66L, we studied its alkaline transition. This transition has been investigated in mammalian Mbs where dissociation of one proton equivalent converts the bound water to hydroxide at alkaline pH [33]. The absorption spectrum of the AHb2 H66L mutant in the ferric form was pH sensitive in the range pH 5.3–11 (Figure 2A). Changes in the absorption wavelength of the Soret band of the acidic and alkaline forms of the protein were normalized, and the molar fraction of the alkaline population was calculated and plotted against pH (Figure 2B). The apparent p*K* value of the alkaline transition was calculated to be 7.3 ± 0.08. This pH-dependent behavior of the AHb2 H66L mutant was not observed in the wt AHb2 (not shown), indicating that replacement of His66 is the origin of the alkaline transition. This acidic-alkaline transition, which has also been observed in human Ngb mutant H64V [34] and murine Ngb H64L [35], is well known from many other ferric heme proteins. The p*K* in AHb2 H66L is similar to the one observed in Ngbs [35]. Above pH 9, the absorption at 409 nm decreases slightly, which signals a protonation with p*K* ≈ 10. This suggests that the sixth heme ligand of ferric alkaline H66L is not a hydroxide, but instead, an endogenous group that is pH-labile. This transition was much more evident in the deoxy spectra.

At low pH, the deoxy form of AHb2 H66L was characterized by a broad Soret band, with a maximum at 422 nm and a pronounced shoulder at 436 nm (Figure 2C). The second derivative spectrum in Figure 2C (inset) displays two minima, indicating that the Soret consists of two peaks. We assigned the shoulder at 436 nm to the pentacoordinate species. The prominent species at 422 nm is characteristic of hexacoordinate low-spin heme. The spectral region between 500 and 600 nm exhibits a broad absorption feature at approximately 560 nm. The spectra are pH-independent between pH 5.5 and 8.

At more alkaline pH, the spectra changed dramatically (Figure 2C): the Soret band becomes much narrower, and the shoulder at 436 nm completely disappears. Moreover, two narrow and tall bands at 527 and 557 nm arose from the broad 560-nm absorption. These changes clearly signaled the appearance of a new low-spin heme iron species. The absorbance changes at 557 nm were plotted against pH (Figure 2B). The apparent p*K* value of the alkaline transition in ferrous-deoxy H66L was calculated to be 10 ± 0.2. This behavior suggests that an amino acid near the active site deprotonates and binds to the heme iron. This process was also observed in Ngb [34,35]. We can suppose that these changes are associated with ligation of the Lys69 (E10) amino group to the heme iron after deprotonation, which takes place with a p*K* of ≈10. To verify this assignment we produced a double mutant AHb2 H66L-K69L and examined its spectroscopic features. Interestingly, consistent with our hypothesis, drastic changes at high pH were absent in the spectra of ferrous-deoxy AHb2 H66L-K69L (Figure 2D) (Table 1).

### 2.2. Nitrite Reductase Activity of AHbs Mutants

**Figure 3:**
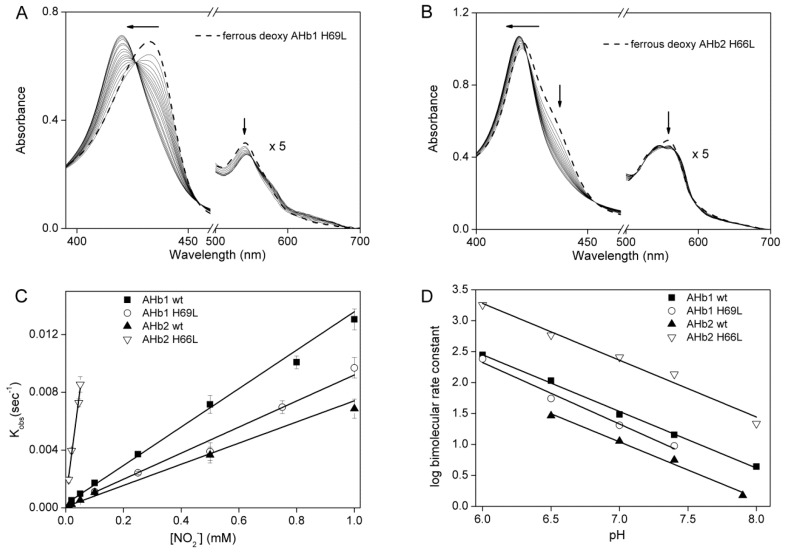
Reaction of ferrous-deoxy AHbs with nitrite. Spectral changes observed for (**A**) ferrous-deoxy AHb1 H69L in the presence of 50 µM sodium nitrite collected at 66-s intervals and (**B**) ferrous-deoxy AHb2 H66L in the presence of 10 µM sodium nitrite at 23-s intervals. All AHbs were treated with 2 mM sodium dithionite. Spectra are expanded by a factor of five from 500 to 700 nm. The arrows indicate the time dependent shift and decrease in spectral bands of ferrous-deoxy AHb1 H69L and AHb2 H66L following the addition of nitrite; (**C**) Plot of *k*_obs_ *versus* nitrite concentration for the reaction of each AHb with nitrite at pH 7.4 and 25 °C; (**D**) Effect of the pH on the nitrite reduction rate by AHbs.

The reaction of ferrous-deoxy AHb1 H69L and AHb2 H66L with nitrite was studied to determine the effect of distal His mutations on the AHbs nitrite reduction rate. Figure 3A,B shows the spectral changes observed for ferrous-deoxy AHb1 H69L and ferrous-deoxy AHb2 H66L, respectively, in the presence of sodium nitrite and 2 mM sodium dithionite. Following the addition of nitrite, we observed a shift in the spectrum from a ferrous-deoxy form to a ferrous-nitrosyl form with clear isosbestic points. The time course of spectral changes at 423 nm for AHb1 H69L and at 436 nm for AHb2 H66L were fit to a single-exponential equation to find the observed rate constant (*k*_obs_). Inclusion of dithionite helped to avoid the formation of the oxygenated nsHb species and rapidly reduced the resulting ferric AHb1 H69L and AHb2 H66L forms to the deoxy forms (Equations (1) and (2)). In the absence of sodium dithionite, two molecules of deoxy-Hb form one ferrous-nitrosyl and one ferric species; however, we always performed the nitrite reduction in excess of dithionite, where ferric-Hb is reduced back to the ferrous-deoxy species and, thus, could react again as described in Equation (2). Therefore, one molecule of deoxy-AHb forms one molecule of ferrous-nitrosyl Hb with a rate that is the same as the deoxy-Hb consumption rate. It has been shown that nitrite is not effectively reduced to NO by dithionite (1–5 mM) [9]; thus, ferrous-nitrosyl-AHbs results from the reduction of nitrite mediated by deoxy-AHb.

(1) $${\text{Fe}}^{2+}+{\text{ NO}}_{2}^{-}+H^{+}\to {\text{Fe}}^{3+}+{\text{NO}}^{\cdot }+{\text{OH}}^{-}$$

(2) $${\text{Fe}}^{2+}+{\text{NO}}^{\cdot }\to {\text{Fe}}^{2+}-\text{NO}$$

Under the pseudo-first order experiment conditions, the bimolecular rate of the nitrite reduction can be extracted from the linear fit of the obtained *k*_obs_ *versus* nitrite concentration. We found that the rate of deoxy-AHb conversion to nitrosyl-AHb was faster for the AHb2 H66L mutant, which showed a bimolecular rate of 171.90 ± 9.00 M^−1^·s^−1^, approximately 25-fold higher than for the AHb2 wt (7.3 ± 0.5 M^−1^·s^−1^) (Figure 3C). An analogous effect was observed with human Ngb in which the mutation of the distal His with either a Leu or Gln residue resulted in about a 2000-fold increase of the nitrite reductase reaction rate. Interestingly, we did not observe the same effect with AHb1 H69L protein, which is instead characterized by a lower nitrite reductase rate (10.6 ± 0.86 M^−1^·s^−1^) than AHb1 wt (13.3 ± 0.40 M^−1^·s^−1^).

As spectroscopic analysis of the AHb2 H66L-K69L double mutant indicated a pentacoordinate species, we were interested in measuring its nitrite reductase activity. Unfortunately, our attempts to perform nitrite reductase reaction with this mutant were unsuccessful, as the values obtained were not reproducible and reliable. This might be ascribed to an observed partial instability of the protein which, with time, showed features characteristic of partial denaturation. A similar phenomenon was also observed with the double mutant H64L-K67L of murine Ngb at pH >8.5 [35] and with H46L-Q43L of Synechocystis Hb [36], which was ascribed to the fact that the double mutation created a highly unstable distal pocket.

### 2.3. pH-Dependent Nitrite Reductase Activity of AHb Mutants

We next examined the pH dependence of the nitrite reductase activity by AHb wt and the mutant proteins in the pH range of 6–8 (Figure 3D). By plotting the log of the bimolecular rate constants *versus* pH, we obtained a line with a slope of 0.92 ± 0.03, 0.85 ± 0.07, 1.01 ± 0.08 and 1.02 ± 0.09 for AHb1 wt, AHb2 wt, AHb1 H69L and AHb2 H66L, respectively. These values are in broad agreement with the value of one that was predicted according to Equations (1) and (2). We conclude that deoxygenated AHb1 and AHb2 reduce nitrite via the transfer of an electron and a proton to form NO, similar to that hypothesized for Ngb and other mammalian Hbs [10,31].

### 2.4. Detection of NO Gas by Chemiluminescence

**Figure 4:**
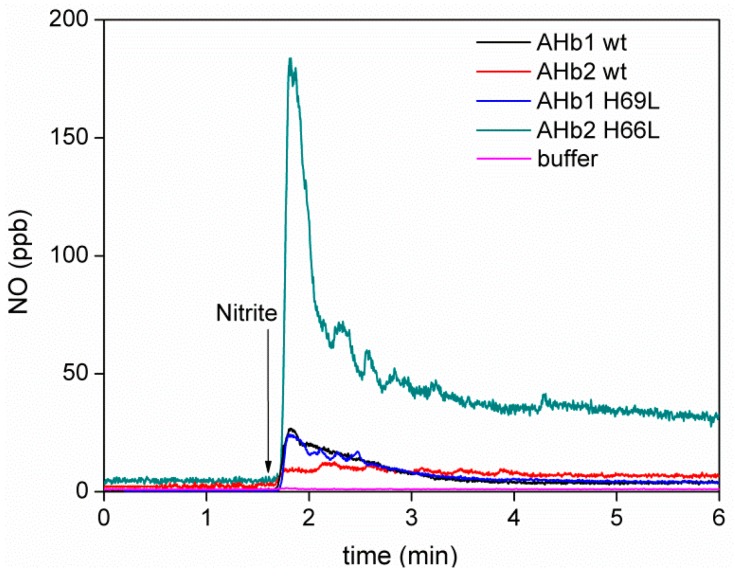
Formation of NO gas during the reduction of nitrite by deoxy AHbs. Typical chemiluminescence NO traces of the NO-detection system after the addition of 2 mM nitrite (indicated by the arrow) into buffer alone (magenta), 10 µM deoxy AHb1 wt (black), AHb2 wt (red), AHb1 H69L (blue) and AHb2 H66L (green). Chemiluminescence traces are representative of three or more experiments.

Although in our *in vitro* conditions, deoxy-AHbs can recapture the NO, we next used a chemiluminescence NO analyzer to provide qualitative information on possible free NO gas release. Figure 4 shows the generation of NO in gas phase for deoxy AHb1 wt, AHb2 wt, AHb1 H69L and AHb2 H66L mutants.

Despite relatively fast autoxidation in the presence of O_2_, we were able to obtain reduced AHbs in anaerobic conditions using the special enzymatic system developed by Hayashi (see the Materials and Methods, Section 4.3) [37]. This system reduces the AHbs more slowly compared to dithionite (it takes 2–3 h for the oxidized AHbs to become reduced compared to the immediate effect of dithionite). As ferric AHbs will not become reduced as fast as observed with dithionite, free NO gas could be measured. The absorption spectra of deoxy AHbs obtained by the Hayashi system fully corresponded to that obtained in the presence of sodium dithionite. Interestingly, the amount of NO released for AHb2 H66L mutant was significantly higher than that for AHb2 wt. However, the same was not true with AHb1 H69L, where the amount of NO detected was very similar to AHb1 wt.

### 2.5. Reaction of Nitrosyl AHbs with Peroxynitrite and H_2_O_2_

**Figure 5:**
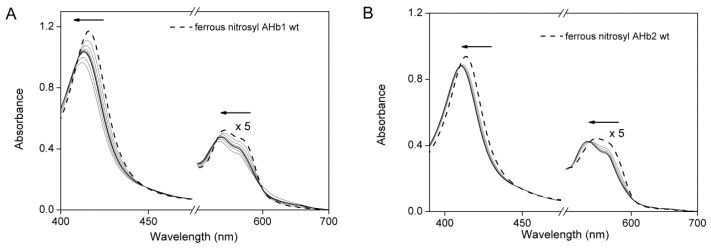
Reaction of nitrosyl-AHbs with peroxynitrite. Spectral changes associated with the oxidation of ferrous-nitrosyl AHb1 wt (**A**) and ferrous-nitrosyl AHb2 wt (**B**) (dashed line) in the presence of 20 μM peroxynitrite. Spectra were collected at 3-min intervals. The arrows indicate the time dependent shift in spectral bands of ferrous-nitrosyl AHb1 wt and AHb2 wt upon peroxynitrite addition. Ferric-AHbs (solid line) are generated at the end of the reaction, and their spectra are comparable to the reference spectra of ferric-AHb. The region from 500 to 700 nm is expanded by a factor of five.

The reaction of nitrite with plant Hbs is potentially physiologically significant and may lead to the formation of ferrous-nitrosyl species, as we have demonstrated herein. It has been suggested that nitrosyl-hemoglobin (Fe^+2^–NO) may represent a form of stabilized NO [38,39] and may be responsible for NO delivery to the various tissues, where it participates in a variety of biologically-relevant reactions. We thus wanted to test if NO could be released from ferrous nitrosyl-AHb upon exposure of oxidizing agent, such as peroxynitrite (ONOO–). As shown in Figure 5, upon the addition of peroxynitrite, the characteristic spectra of ferrous nitrosyl-AHb at 417/543/565 nm (AHb1 wt) and 416/555 nm (AHb2 wt) converted to their respective ferric form at 412/536/565 nm (AHb1 wt) and 410/534/562 nm (AHb2 wt). We observed the formation of ferric-AHb species at the end of the reaction with the characteristic spectrum of pure oxidized AHbs.

Previous research supports our observation on the release of NO from nitrosyl-Hbs upon treatment with oxidizing agents, such as peroxynitrite [38] or potassium ferricyanide [40]. Herold *et al.* [38] explained that the release of NO from ferrous-nitrosyl Hbs upon oxidation with peroxynitrite involves the formation of ferric-nitrosyl Hb as an intermediate. The NO is dissociated from ferric-nitrosyl Hb to give pure ferric-Hbs. A similar behavior was seen upon the treatment of ferrous-nitrosyl AHbs with hydrogen peroxide (data not shown).

### 2.6. Sequence Analysis of AHbs and Homology Model of AHb2

**Figure 6:**
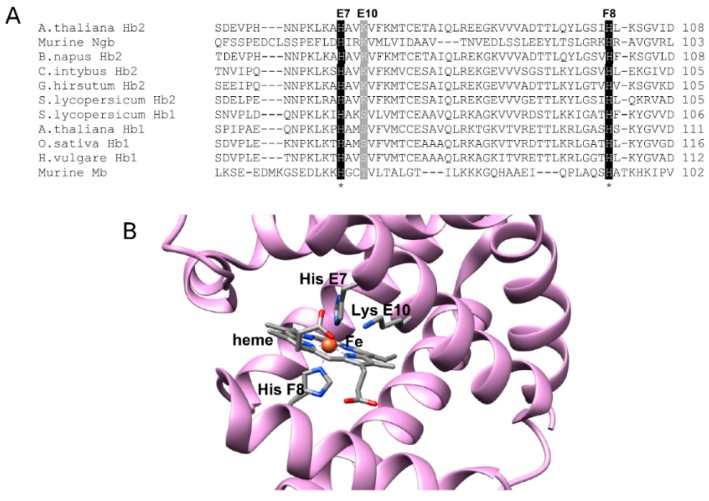
Multiple sequence alignment from different globins and homology-based molecular modeling of AHb2. (**A**) The black boxes indicate the residues of the heme pocket in position E7 (distal His) and F8 (proximal His), while the gray box indicates residue in position E10; (**B**) Structural model of AHb2 wt. Secondary structure elements are represented by cartoons. Residues in the binding cavity are shown together with the heme group. Atoms are depicted in stick representation and colored using the color code: carbon, oxygen, nitrogen and iron in gray, red, blue and orange, respectively.

Amino acid sequence alignments with other hexacoordinate Hbs (Figure 6A) indicate that AHb2 has a Lys residue at position E10, while AHb1 has a Ser residue. Among the distal residues, LysE10 has been found to be functionally important to Ngb [34,41]. This Lys residue is involved in a network of *H*-bonding comprising the heme propionate side chain, which results in limited access of the heme iron to ligands [42]. As demonstrated by the double mutant AHb2 H66L-K69L, it would seem reasonable that Lys69 in AHb2 is situated close to the heme iron, enabling ligation at alkaline pH in H66L mutant with an increase in pocket polarity, which might explain the increase in nitrite reductase activity in AHb2 H66L, as shown for murine Ngb [35]. A molecular model of AHb2 (Figure 6B), built using the structure of the murine Ngb as a template (PDB Accession Code 1Q1F) that was co-crystalized with the heme, seems to be consistent with this assignment.

## 3. Discussion

*In vitro* nitrite reductase activity by Hbs under anoxia has been demonstrated in animals, cyanobacteria and plants [7,8,9,10]. Our experiments, in addition to confirming that under conditions of anoxia, *Arabidopsis* Hb1 and Hb2 can function as nitrite reductases and that the reaction rates increase linearly as [H^+^] increases, reveal that comparable HisE7Leu mutations in AHb1 and AHb2 took opposite directions in terms of nitrite reductase activity. Replacement of AHb2 distal His with Leu leads to a significant increase in the nitrite reductase rate (~25-fold), while the same mutation in AHb1 leads to a small decrease in reactivity. Consistent with the observed nitrite reductase rates, NO released from AHb2 H66L was significantly higher compared to AHb2 wt, while NO release was slightly decreased in AHb1 H69L compared to AHb1 wt.

In hexa-coordinate Hbs, such as AHb1 and AHb2, distal HisE7 reversibly binds to heme iron as a sixth ligand and plays an important role in regulating external ligand migration and binding affinity to the heme iron [26]. In the absence of ligand, AHb1 exists as an equilibrium between the pentacoordinated and hexacoordinated forms, while AHb2 is fully hexacoordinated [28].

There are at least two features of the AHb2 H66L mutant that may contribute to its high catalytic nitrite reduction. First, hexacoordination in the AHb2 H66L mutant augments the rate of both heme reduction and electron transfer through decreasing the activation energy associated with the oxidation state alteration [43]. This is because its heme iron remains in low-spin in both the ferrous-deoxy and ferric oxidation states. On the contrary, the replacement of distal His in AHb1 results in a stable pentacoordinate high-spin species in both ferric and ferrous-deoxy form. Second, hexacoordination could prevent nitrite from reacting with the ferric form and therefore acts as a mechanism to ensure that nitrite catalysis continues in the reductive direction.

Previous resonance Raman studies on CO complexes of ferrous AHb1 and AHb2 have provided solid support that HisE7 is directly involved in assisting heme-bound CO via a hydrogen bond in AHb1 wt, while in AHb2-CO, the non-polar heme pocket environment suggests that distal His is distant from the bound ligand [26,30]. This difference may have functional relevance, as previous studies found that mutated heme proteins in which the distal cavity has only limited polarity (e.g., myoglobin H64L [44]) are associated with a reduction in the activity of nitrite reductase. Thus, the lower bimolecular rate value of AHb1 H69L and AHb2 compared to AHb1 could result from the different polarity of the distal cavity. Interestingly, amino acid sequence alignments of different plant hexacoordinated globins showed that class 1 nsHbs have a Ser residue in position E10, while class 2 plant Hbs display a Lys residue in this position. Our spectroscopic data on the AHb2 H66L mutant and the AHb2 H66L-K69L double mutant, together with a molecular model prediction, suggested that Lys69 acts as a second endogenous ligand at high pH when it becomes deprotonated, allowing us to conclude that in AHb2 the heme iron is highly reactive, and at alkaline pH, the lysine side chain competes with the exogenous ligand and the His66 for the sixth coordination, as reported for murine Ngb [35] and human Ngb [34]. This might explain the increase in nitrite reductase activity in AHb2 H66L.

It has been proposed that the polarity of the heme pocket may regulate the efficient NO detoxification from truncated Hb by *M. tuberculosis*; generation of nitrate from NO and oxygen at the heme pocket may be facilitated by the polar environment of iron bound oxygen. The cavities’ system may in turn provide easy movement of the partner molecules, such as NO, from the solvent to the distal heme cavity [45,46]. After accumulation of nitrate generated from NO and oxygen, the hydrophobic tunnel may promote rapid removal of the polar product (nitrate) from the reaction cavity to the solvent space [46]. Similar mechanisms may be at work in AHb’s nitrite reduction activity. Increased polarity may allow efficient movement of polar product, nitrite, at the distal heme pocket, which may result in an increased rate of nitrite reduction.

It is also possible that the differences in nitrite reactivity for AHb2 and AHb1 (and the different requirements for ligand hydrogen binding) could be related to differences in the preferred nitrite-binding mode. This in turn may be related to the electronic properties of heme. In the ferric H64V-nitrite structure of horse heart myoglobin, mutation of the distal His with Val allows the nitrite to adopt the nitro (*N*-binding) form instead of the nitrito (*O*-binding) form in the wt protein, which alters the nitrite reduction rate. Interestingly, the reintroduction of a residue able to form an *H*-bond by preparing the H64V/V67R double mutant restores the *O*-binding mode of nitrite and reactivity [44]. These concepts warrant further studies; resolving the crystal structure of the nitrite-bound ferric nsHbs complex could provide definitive experimental insight into the nature of this rather complex nitrite-heme interaction. Therefore, the polarity of the distal pocket, the system of cavities and tunnels and the protein dynamics all may play an important role in regulating the nitrite reductase activity by AHbs. Indeed, as exhaustively discussed by Tejero *et al.* [47], many factors, such as heme accessibility, redox potential, histidine protonation, polarity of the distal cavity and nitrite binding mode, have been related to rate of nitrite reductase.

Moreover, other residues in the distal pocket could affect the nitrite reduction. In this scenario, the residue PheB10 (conserved both in AHb1 and AHb2) is of particular interest, for its crucial role in affecting hexacoordination and the autoxidation rate [48,49]. In particular, previous studies on AHb1 showed that PheB10, which is structurally near to HisE7, appears to have a stabilizing effect on hexacoordination (the equilibrium constant for hexacoordination (*K*_H_) was ≈0.5 for the PheB10Leu mutant and ≈1.6 for wt AHb1), reflecting the key role of this residue in the equilibrium between hexa and penta species in *Arabidopsis* Hb1 [49]. In contrast, for rice Hb1, it was shown that mutation of PheB10 with leucine likely favors the formation of a purely hexacoordinated species [48,50]. Spectral data for CO complexes suggested that AHb1 PheB10 likely participates in stabilization of the heme-bound ligand by indirectly facilitating a hydrogen bond with HisE7 [49]. Moreover, neighboring PheB10 helps the distal His in stabilizing bound ligand, as it provides further protection against solvation; indeed, the accessibility of the heme pocket to OH^−^ seems to be around 100-fold higher for PheB10Leu mutant than for wt [51]. With regards to the subtle relation between HisE7 and PheB10, which is vital in modulating a balance between the penta- and hexa-coordinated forms of AHb1 wt, the exploration of the role of PheB10 in nitrite reduction activity of AHb1 and AHb2 seems to be fundamental and deserves future investigations.

We also observed that NO can be released from AHbs (Fe^+2^–NO) by peroxynitrite. Previous research supports our observation on the release of NO from nitrosyl-Hbs upon treatment with oxidizing agents, such as peroxynitrite [38] or potassium ferricyanide [40]. Herold *et al.* [38] showed that the release of NO from ferrous-nitrosyl Hbs upon oxidation with peroxynitrite involves the formation of met-nitrosyl Hb as an intermediate. NO is dissociated from met-nitrosyl Hb to give pure ferric Hbs. Since the Fe^+3^–NO is bound only weakly (*K*_d_ in mM range), after oxidation of the iron center (the conditions of our experiment), over 90% of NO is released. Peroxynitrite-treated ferrous-nitrosyl AHb releases NO, which then could inactivate mitochondrial respiration [52,53]. NO regulates the oxygen gradient by competitively inhibiting cytochrome c oxidase in mitochondria [52]. Under such conditions, external NADH or NADPH is oxidized by plant mitochondria, and limited energy is retained for ATP synthesis, complementing glycolytic ATP production [52]. This process can be seen in another way, in that AHb is a detoxifying peroxynitrite, a potent oxidizing and nitrating agent, which is generated at high levels during hypoxia and contributes to plant cell damage and lipid peroxidation [54]. Mb and Hb have been shown to scavenge peroxynitrite and, thus, protect cells against oxidative and nitrosative stress [55]. This process may be physiologically significant in cells under stress conditions.

Taken together, the results presented herein further support the idea that involvement in NO metabolism is a key component in the function of nsHbs. Even if it is well known that under hypoxia a main function of class 1 plant Hbs is to scavenge NO by reacting with NO in their oxygenated form to produce nitrate [25], under conditions of extreme hypoxia or anoxia, plant class 1 Hbs may also work as nitrite reductases by reducing nitrite to NO efficiently, an activity that accelerates at acidic pH [8,9]. Plants often face stress conditions that can lead to oxygen deprivation, such as during flooding and waterlogging. Nitrate or nitrite concentrations can increase up to very high concentrations (mM) [56,57], and the pH in plant cells can also decrease significantly [58].

High nitrite concentrations also lead to the accumulation of ROS [59]. Thus, in conditions where soil is poorly oxygenated, and in particular for species that are water submerged, pH-dependent reduction of nitrite through deoxygenated nsHbs is a reasonable pathway to generate NO in hypoxia/anoxia.

As for other plant Hbs, it is possible that AHb1 and AHb2 are able to regulate NO levels in plant cells by acting as NO dioxygenase and/or nitrite reductase and indirectly controlling the multitude effects that NO has on overall plant physiology considering the availability of ambient oxygen. Interestingly, *Hb* gene expression, in particular AHb1, was substantially augmented in flooded roots from *Arabidopsis*, in agreement with the possibility that plant Hbs are induced by reduced oxygen diffusion occurring during root hypoxia [60]. *In vivo* experiments showed that, under hypoxia, the presence of nsHbs was associated with a decrease in NO levels in *Arabidopsis* [60], supporting NO dioxygenase activity, whereas in barley, the presence of nsHbs was associated with slightly increased NO levels in anoxic tissues [61], supporting the view that nitrite reductase activity of nsHbs may occur *in vivo* under such conditions.

Although these data highlight and confirm the role of nsHbs in influencing cell signaling and metabolism by modulating the levels of NO, the available *in vivo* studies concerning cellular interactions and regulation of plant metabolism by AHb1 and AHb2 are presently inadequate to obtain a more in-depth understanding of their physiological roles; in this regard, additional studies *in vivo* will be needed to elucidate the precise function of these proteins.

## 4. Materials and Methods

### 4.1. Reagents and AHb Preparations

All reagents were purchased from Sigma, unless otherwise indicated. Sodium dithionite, potassium ferricyanide and sodium nitrite stocks were dissolved in buffer shortly before use. When needed, solutions were degassed with argon and kept protected from light. AHb1, AHb2 and AHb1 H69L were cloned, expressed and purified as previously described [26,49]. AHb2 H66L and AHb2 H66L-K69L mutants were made on the wt construct pGEM-AHb2 using the QuikChange II mutagenesis kit (Stratagene, La Jolla, CA, USA). The conditions for expression and purification of the mutants were as described for the wt protein [26].

The extinction coefficient spectra of AHb in the ferrous-deoxy, ferric and ferrous-nitrosyl forms were recorded using pure solutions of each species. AHb was diluted into 0.1 M phosphate buffer pH 7.4 to a known final heme concentration, which was calculated by using the pyridine-hemochromagen method [62]. Ferric AHb was determined after complete oxidation of the protein by potassium ferricyanide and elimination of excess ferricyanide using Vivaspin concentrating filters (Sartorius AG, Göttingen, Germany). The deoxy form of AHb was obtained by adding sodium dithionite (2 mM final concentration) and pure ferrous-nitrosyl AHb was prepared by adding (±)-(*E*)-4-methyl-2-[(*E*)-hydroxyimino]-5-nitro-6-methoxy-3-hexeneamide (NOR1, Calbiochem, San Diego, CA, USA) or 2-(*N*,*N*-diethylamino)-diazenolate-2-oxide sodium salt  (DEA/NO), which are synthetic NO donors that release the indicated amounts of NO.

pH titration of ferric and ferrous-deoxy AHb2 H66L and ferrous-deoxy AHb2 H66L-K69L was performed by dissolving the protein in 100 mM sodium phosphate/citrate (pH 4–6), sodium phosphate (pH 6.2–8.6) and sodium carbonate buffer (pH > 8.6) to a final concentration of ~5 μM. The absorbance changes at 558 nm for ferrous-deoxy AHb2 H66L and at 411 for ferric AHb2 H66L are plotted as a function of pH after rescaling from 0 to 1 using the following Equation (3) as in [35]:

(3) $$c_{+}\;\left(\text{pH}\right)=1\;-\;c_{0}\left(\text{pH}\right)=\;\frac{1}{1+\;10^{n\left(\text{pH}-\text{pK}\right)}}$$

where $c_{+}$ and $c_{0}$ are the fractional populations of the protonated (acid) and deprotonated (base) species, respectively.

### 4.2. Reaction of AHbs with Nitrite

All reactions were carried out with excess sodium dithionite (2 mM) in 0.1 M phosphate buffer at controlled pH and at room temperature using 1-cm sealed quartz cells. The presence of sodium dithionite in the reaction mixture eliminated O_2_ and converted ferric AHb to the ferrous-deoxy AHb without reacting with nitrite [63]. Reaction kinetics were monitored by visible absorption spectroscopy using a JASCO V-560 spectrophotometer(Jasco Europe, Lecco, Italy) under pseudo-first order conditions in the presence of an excess of nitrite. Concentrations of single species during reactions (deoxyheme and iron-nitrosyl-heme) at each time point were determined by least squares spectral deconvolution of the visible absorption spectrum (software Origin Pro 8, Origin Lab Corporation, Northampton, MA, USA) into standard reference spectra [64]. The effect of pH was studied by reacting ferrous-deoxy AHb and nitrite in phosphate buffer adjusted to the desired pH values.

### 4.3. NO Gas Detection by Chemiluminescence

The NO signal was detected using a chemiluminescence NO detector (CLD 88et, Ecophysics, Duernten, Switzerland). Deoxy AHb proteins (final concentration 10 µM) were obtained by reduction in the presence of the Hayashi enzymatic system, which employs NADP, glucose 6-phosphate and glucose-6-phosphate dehydrogenase as a system that generates NADPH and ferredoxin and ferredoxin-NADP reductase as an electron-mediating system [37], similar to the artificial reduction system already used for other hexacoordinated hemoglobins [65]. The reactions were carried out in 1 mL of degassed 0.1 M phosphate buffer pH 7.4 containing 2 mM degassed sodium nitrite in a vessel purged with inert nitrogen. Once a stable line was obtained, deoxy AHb was added to start the reaction.

### 4.4. Release of Nitric Oxide from Nitrosyl-AHb by Oxidizing Agents

AHb proteins (10 μM) were reduced as described above, and ferrous-nitrosyl AHb was prepared by adding excess of nitrite (2 mM). Pure nitrosyl AHb was formed at the end of the reaction and confirmed by absorbance spectroscopy. The remaining sodium dithionite and nitrite were removed by Vivaspin quick filtration, and spectra were again recorded to verify the presence of nitrosyl AHb species. To test the oxidation of ferrous-nitrosyl AHb to ferric AHb with the release of NO, two physiologically-relevant oxidants were chosen, namely peroxynitrite and hydrogen peroxide. Ferrous-nitrosyl AHb1 wt (10 μM) was mixed with peroxynitrite (10 µM) and hydrogen peroxide (100 µM).

### 4.5. Statistical Analysis

Each experiment was performed at least three times, and reported values are representative of two or more independent experiments using different batches of protein purified separately. Data were analyzed using Origin 8.0 software (OriginLab Corporation, Northampton, MA, USA) and expressed as the mean ± standard error.

### 4.6. Sequence Alignment and Homology Modeling

Sequence retrieval of the entire family was performed from the UNIPROT database (http://www.uniprot.org/). The family of curated sequences was then used to build up the multiple sequence alignment by using the Clustal Omega program [66,67]. The AHb2 sequence was then funneled through the SwissModel web-server for template search and model generation. In particular, among the chosen templates, we considered the structure of the murine Ngb as a template (PDB Accession Code 1Q1F) that was co-crystalized with the heme. Target and template share a sequence identity of about 26%. The model was afterwards optimally superimposed onto the structure of murine Ngb by using the Chimera program [68] in order to maintain the chemical features in the structural alignment. We have thus transferred the coordinates of the heme group into AHb2’s binding cavity.

## 5. Conclusions

Here, we have examined the effect of distal histidine residues on heme iron coordination, NO production and nitrite reductase activity of AHb1 and AHb2. Replacement of the distal histidine by leucine leads to a stable five-coordinated geometry in AHb1, while in AHb2 results in a hexacoordinated low-spin species with Lys69 as the sixth ligand. Interestingly, the AHb2 mutant reduces nitrite to NO approximately 25-times faster than the AHb2 wt, whereas the same mutation in AHb1 slightly slows the reaction. Although these data highlight the pronounced differences in the active site properties of AHb1 and AHb2, to date, it is not possible to explain the mechanisms regulating nitrite reductase activity by these proteins since many factors (heme accessibility, redox potential, histidine protonation, polarity of the distal cavity, protein dynamics and nitrite binding mode) have been related to nitrite reductase rates. Importantly, our experiments confirm the relevant role of plant Hbs in NO metabolism via NO dioxygenase and/or nitrite reductase activity, although this role needs to be further elucidated by additional studies *in vivo*.

## Figures and Tables

**Table 1:** Peak positions of the absorption spectra of AHbs variants in different oxidation states for the different pH regions.

AHb Proteins	Ligation State	pH	p*K*	Soret	Visible Bands
				*nm*	*nm*
**AHb1 H69L**					
Ferric	Five-coordinate	6–9		390	504/637
Ferrous-deoxy	Five-coordinate	6–9		435	540/569
**AHb2 H66L**					
Ferric	Five/six-coordinate	6	7.2	404	500/631
		8		408	532/562
		11	10	409	535/568
Ferrous-deoxy	Five/six-coordinate	6		422/436	560
		11	10	422	527/557
**AHb2 H66L-K69L**					
Ferric	Five-coordinate	6–11		397	498/624
Ferrous-deoxy	Five-coordinate	6–11		420/436	538/560

## Abbreviations

Hb	Hemoglobin
AHb	*Arabidopsis thaliana* hemoglobin
Ngb	Neuroglobin
NO	Nitric oxide
NO_2_^−^	Nitrite
ROS	Reactive oxygen species
*k*_obs_	Observed rate constant
*k*_d_	Dissociation rate constant
E7	Seventh amino acid at E-helix in Hb
Fe^2+^	Ferrous
Fe^3+^	Ferric
